# Pitfalls of improperly procured adjacent non-neoplastic tissue for somatic mutation analysis using next-generation sequencing

**DOI:** 10.1186/s12920-016-0226-1

**Published:** 2016-10-19

**Authors:** Lei Wei, Antonios Papanicolau-Sengos, Song Liu, Jianmin Wang, Jeffrey M. Conroy, Sean T. Glenn, Elizabeth Brese, Qiang Hu, Kiersten Marie Miles, Blake Burgher, Maochun Qin, Karen Head, Angela R. Omilian, Wiam Bshara, John Krolewski, Donald L. Trump, Candace S. Johnson, Carl D. Morrison

**Affiliations:** 1 Department of Biostatistics and Bioinformatics, Roswell Park Cancer Institute, Buffalo, NY USA; 2 Center for Personalized Medicine, Roswell Park Cancer Institute, Buffalo, NY USA; 3 Department of Cancer Genetics, Roswell Park Cancer Institute, Buffalo, NY USA; 4 Department of Medicine, Roswell Park Cancer Institute, Buffalo, NY USA; 5 Department of Pathology, Roswell Park Cancer Institute, Buffalo, NY USA; 6 Department of Pharmacology and Therapeutics, Roswell Park Cancer Institute, Buffalo, NY USA; 7 Inova Dwight & Martha Schar Cancer Institute, Falls Church, VA USA

**Keywords:** Somatic mutations, Tumor contamination, Adjacent normal tissues

## Abstract

**Background:**

The rapid adoption of next-generation sequencing provides an efficient system for detecting somatic alterations in neoplasms. The detection of such alterations requires a matched non-neoplastic sample for adequate filtering of non-somatic events such as germline polymorphisms. Non-neoplastic tissue adjacent to the excised neoplasm is often used for this purpose as it is simultaneously collected and generally contains the same tissue type as the neoplasm. Following NGS analysis, we and others have frequently observed low-level somatic mutations in these non-neoplastic tissues, which may impose additional challenges to somatic mutation detection as it complicates germline variant filtering.

**Methods:**

We hypothesized that the low-level somatic mutation observed in non-neoplastic tissues may be entirely or partially caused by inadvertent contamination by neoplastic cells during the surgical pathology gross assessment or tissue procurement process. To test this hypothesis, we applied a systematic protocol designed to collect multiple grossly non-neoplastic tissues using different methods surrounding each single neoplasm. The procedure was applied in two breast cancer lumpectomy specimens. In each case, all samples were first sequenced by whole-exome sequencing to identify somatic mutations in the neoplasm and determine their presence in the adjacent non-neoplastic tissues. We then generated ultra-deep coverage using targeted sequencing to assess the levels of contamination in non-neoplastic tissue samples collected under different conditions.

**Results:**

Contamination levels in non-neoplastic tissues ranged up to 3.5 and 20.9 % respectively in the two cases tested, with consistent pattern correlated with the manner of grossing and procurement. By carefully controlling the conditions of various steps during this process, we were able to eliminate any detectable contamination in both patients.

**Conclusion:**

The results demonstrated that the process of tissue procurement contributes to the level of contamination in non-neoplastic tissue, and contamination can be reduced to below detectable levels by using a carefully designed collection method. A standard protocol dedicated for acquiring adjacent non-neoplastic tissue that minimizes neoplasm contamination should be implemented for all somatic mutation detection studies.

**Electronic supplementary material:**

The online version of this article (doi:10.1186/s12920-016-0226-1) contains supplementary material, which is available to authorized users.

## Background

Cancer is a genetic disease largely caused by somatic mutations [1]. Identifying somatic mutations provides fundamental insights into the development, progression, and personalized treatment of cancer. Recent advances in next-generation sequencing (NGS) provide a very sensitive means for detecting somatic mutations in cancer, but variant calling improves when matched non-neoplastic tissue is sequenced in parallel to filter out non-somatic events, such as germline polymorphisms or “normal” transcripts [2]. For solid neoplasms, the most commonly used non-neoplastic sources are blood, saliva, or non-neoplastic tissue adjacent to the neoplasm [2–9]. Compared with blood or saliva, the adjacent non-neoplastic tissue is conveniently collected during the same surgical procedure, and often consists of the same cell type as the neoplasm. However, we and others have frequently observed the presence of low level somatic mutations in these non-neoplastic adjacent tissues in prior studies, which imposes additional challenges in distinguishing somatic mutations from germline polymorphisms (Additional file 1: Figure S1) [10].

The presence of somatic mutations in adjacent non-neoplastic tissue may be explained by a “field effect” or by simple contamination by neoplastic tissue. The concept of the carcinogenesis field effect is not new, but has been recently re-defined as epithelium with “normal” morphology and genetic alterations that can result in the development of an overt malignant neoplasm [11, 12]. There is considerable literature supporting the field effect concept in malignancies from multiple organ systems [13–20]. However, for breast cancer, in particular, at least one report indicates that microscopically identifiable malignant cells, attributed to tumor cell contamination, are often found in adjacent non-neoplastic breast tissue [21]. Such contamination of the matched non-neoplastic tissue can limit the sensitivity of a genomic analysis by inadvertently excluding mutations that may be important for neoplasm progression, prognosis, or treatment.

To determine if sample contamination during tissue grossing and procurement is a major cause of the presence of somatic mutations in non-neoplastic tissues, we systematically acquired multiple non-neoplastic tissues using different collection methods around the same neoplasm, and assessed the presence of mutant alleles in non-neoplastic tissues using next-generation sequencing. By comparing the contamination levels of non-neoplastic tissues from different collection methods, we demonstrated in this study that neoplasm contamination can be reduced to below detectable levels by using a carefully designed collection method.

## Methods

### Specimens

The contamination problem was initially observed in a subset of previously sequenced breast cancers where the neoplasm, adjacent non-neoplastic tissue and matched blood were all available (Additional file 1: Figure S1, S2). These specimens were grossed for the general purposes of variant discovery. The grossing procedure was not specifically designed for evaluating contamination problem so that information such as whether clean instruments were used as well as the distance from the neoplasm the non-neoplastic tissue was procured was not available. For further and more detailed analysis of the contamination problem specimens were collected from two breast lumpectomies following a written grossing and tissue procurement protocol (Additional file 2: Supplemental materials: Grossing and tissue procurement procedure for special collections). Grossing was performed by a certified Pathologist Assistant (PA). All patients were consented for specimen collection under an institutional approved IRB protocol. The final diagnosis for both specimens was invasive breast carcinoma. The neoplastic cellularity of the two breast lumpectomies was estimated to be 50 and 70 %, for Patient1 and Patient2, respectively.

### Collection of adjacent non-neoplastic samples

**Fig. 1:**
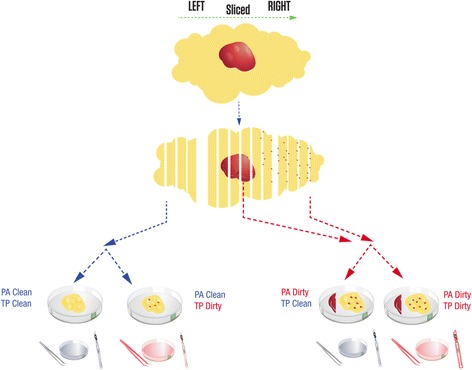
Design of the special collection of non-neoplastic breast tissue. After orienting, the lumpectomies were bread-loafed from left to right using one blade for the entire specimen. Non-neoplastic tissue sectioned before the neoplasm was designated “PA Clean”. Non-neoplastic tissue sectioned after the neoplasm was designated “PA Dirty”. The forceps, scalpel, and Petri dish used to cut the neoplasm were referred to as “dirty” tools (*red*). The forceps, scalpel, and Petri dish that had no contact with the neoplasm were referred to as “clean” tools (*blue*). During the tissue procurement process, samples collected with “clean” tools and “dirty” tools were designated “TP Clean” and “TP Dirty”, respectively. Except for a section of neoplasm, four samples were collected from each lumpectomy: “PA Clean” and “TP Clean” (Clean/Clean), “PA Clean” and “TP Dirty” (Clean/Dirty), “PA Dirty” and “TP Clean” (Dirty/Clean), “PA Dirty” and “TP Dirty” (Dirty/Dirty). Grossly non-neoplastic tissue fragments that had contact with neoplastic tissue have specs of *red* which are reflective of theoretical contamination

The two breast lumpectomies were sectioned from left to right using one blade for the entire specimen (Fig. 1). Grossly non-neoplastic tissue, sectioned before the neoplasm, was designated “PA Clean” (PA: pathologist assistant). Grossly non-neoplastic tissue, sectioned after the neoplasm, was designated “PA Dirty”. Three tissue samples were obtained from each of the two sectioned lumpectomy specimens: a “PA Clean” section, a “PA Dirty” section, and a section of the neoplasm. The “PA Clean” section was placed in one Petri dish, while the “PA Dirty” section and the neoplasm section were placed in a second Petri dish (Fig. 1). Placing the “PA dirty” section and the neoplasm section in the second Petri dish was meant to mimic the way neoplasm and non-neoplasm samples are often allowed to interact during grossing (that is, without significant consideration by the grosser regarding possible microscopic contamination of the non-neoplastic tissues).

Using a clean scalpel and forceps, the “PA Clean” section in the first Petri dish was cut in half. The first piece of the “PA Clean” section was cut in 300–500 mg pieces using clean forceps and scalpel, placed in a cryovial, and snap-frozen in LN_2_. This tissue was referred to as “PA Clean” and “TP Clean” (TP: tissue procurement) or “Clean/Clean”. The second piece of the clean non-neoplastic tissue in the first Petri dish was cut in 300–500 mg pieces using the “dirty” forceps and scalpel used to section the neoplasm in the second Petri dish (see next paragraph), and snap-frozen. Once frozen, the tissue was transferred into a cryovial using the “dirty” forceps. This tissue was referred to as “PA Clean” and “TP Dirty”, or “Clean/Dirty” (Fig. 1).

Using a new set of scalpel and forceps, the neoplasm section in the second Petri dish was cut into 300–500 mg pieces. A neoplasm fragment was snap frozen in a container with liquid nitrogen (LN_2_) and transferred to a cryovial. Using a new set of scalpel and forceps, the “PA Dirty” section (also located in the second Petri dish) was cut in half. The first piece of the “PA Dirty” section was cut in 300–500 mg pieces, placed into another cryovial and snap-frozen. Once frozen, the tissue was transferred into a cryovial using the “clean” forceps. This tissue is referred to as PA “Dirty” and TP “Clean”, or “Dirty/Clean”. The second “PA Dirty” section was cut in 300–500 mg pieces using the “dirty” forceps and scalpel previously used to section the neoplasm fragment (see beginning of this paragraph) and placed directly in the container with LN2. Once frozen, the tissue was transferred to a cryovial using the “dirty” forceps. This tissue was referred to as “PA Dirty” and “TP Dirty”, or “Dirty/Dirty”.

To histologically evaluate the procured tissues, they were removed from storage and frozen sections were cut for the pathologist’s review. For each case, QC slides were cut, stained with H&E, and assessed by a pathologist for the presence of neoplasm or absence of neoplasm. The full grossing procedure is in the Additional file 2: Supplemental Methods Section.

A matched peripheral blood sample was collected peri-operatively. DNA from the frozen breast tissues and the matched peripheral blood were extracted following standard DNA extraction SOPs (Additional file 2: Supplemental methods).

### Whole-exome sequencing

Individual exome capture of each DNA sample followed by single-indexed library generation was carried out using a SureSelect XT All Exon V5 kit (Agilent Technologies, Inc). Three micrograms of genomic DNA from each sample was fragmented to a size range of 150–200 bp followed by end repair, adaptor ligation, and six cycles of PCR amplification. Libraries were purified and validated for appropriate size (225–275 bp) on a 2100 Bioanalyzer DNA1000 chip (Agilent Technologies, Inc.). 750 ng of purified library were then hybridized to the SureSelectXT All exon V5 Capture library for 18 h at 65 °C. The captured regions were then bound to streptavidin magnetic beads and washed to remove any non-specific bound products. The eluted library underwent a second 12 cycle PCR amplification to add sample specific barcodes for multiplexing. Final libraries were purified, validated by a bionanalyzer (250–350 bp), and quantitated using a KAPA qPCR library quantitation kit (Kapa Biosystems). Individual libraries were pooled at equimolar 2 nM final concentration. Each pool was normalized to 10 pM, loaded, and clustered to individual lanes of a HiSeq Flow Cell using an Illumina cBot (TruSeq PE Cluster Kit v3), followed by 2 × 101 PE sequencing on a HiSeq2500 sequencer according to the manufacturer's recommended protocol (Illumina, Inc.).

### Targeted amplicon sequencing

The sequencing libraries were prepared using a two-step PCR method targeting approximately 300 bp surrounding the somatic variants of interest. Primers targeting the genomic regions were designed using Primer3 software (http://bioinfo.ut.ee/primer3-0.4.0/) and forward adaptor sequence 5’-TCGTCGGCAGCGTCAGATGTGTATAAGAGACAG-3’ and reverse adaptor sequence 5’-GTCTCGTGGGCTCGGAGATGTGTATAAGAGACAG-3’ were added to the primers necessary for the second round PCR amplification. The first round PCR utilized a gradient annealing temperature which decreased by 1 °C per cycle from 68 to 58 °C followed by 25 cycles with a 57 °C annealing temperature and 72 °C extension and 94 °C denature steps. Genomic (12.5 ng) DNA was used to amplify each of the target regions. After purification with AMPureXP beads (Beckman Coulter) and analysis on a Bioanalyzer DNA1000 chip (Agilent Technologies, Inc.) to ensure the desired target size, the amplicons from the same sample were normalized and pooled to a total of 15 ng using quantitation data from the Bioanalyzer. The amplicon pool from the first round PCR was amplified with eight cycles of PCR using a Nextera Index Kit (Illumina, Inc.), which uses primers that target the overhang adaptor sequence incorporated during the first round of PCR to add a unique indexed tag to each sample which allows pooling of libraries and multiplex sequencing. Indexed libraries were purified with AMPureXP beads, run on a Bioanalyzer DNA1000 chip to verify desired size distribution, quantitated using a KAPA qPCR library quantitation kit (Kapa Biosystems) and pooled in an equimolar fashion to a final concentration of 4 nM. 2 × 150 cycle sequencing was performed on a MiSeq (Illumina, Inc.), according to the manufacturer’s protocol.

### Somatic mutation detection from neoplasm and the matched blood samples

High quality paired-end reads passing the Illumina RTA filter were aligned to the NCBI human reference genome (hg19) using Burrows-Wheeler Aligner [22]. PCR duplicated reads were marked and removed by using Picard (http://broadinstitute.github.io/picard/). Putative SNVs and Indels were identified by running the variation detection module of Bambino [23]. After initial variant calling, the predicted mutations were further filtered to remove potential false calls: (1) the alternative allele is present in the matched blood sample and the Fisher’s exact test P value not less than 0.05; (2) the mutant alleles are only present in one of the two strands and the Fisher’s exact test *p* < 0.05; (3) the putative mutation occurs at a site with systematically dropped base quality scores, defined as more than 70 % of the mutant reads with Phred quality score reduced by at least 10 at the mutant base compared with the 5’ or 3’ side neighboring bases. All identified mutations were manually reviewed using Bambino viewer (Edmonson, Zhang et al. [23]). The functional effects were predicted by ANNOVAR [24] using RefSeq sequence database downloaded from NCBI on March 22, 2015.

### Assessing contamination in non-neoplastic samples

For each somatic mutation identified from the neoplasm whole-exome sequencing (WES) data, the mutation site was re-sequenced in all matched blood and non-neoplastic adjacent tissue, by either WES or TAS. The NGS reads at the mutation site were extracted from the BAM file and classified by mutation status into three groups: mutant, non-mutant (wild-type or a different allele), and unknown (e.g. low base quality). The mutation statuses of paired reads were consolidated and any read pairs where the forward and reverse reads had conflicting mutant status were excluded from the analysis. The coverage and VAF were calculated based on mutant and non-mutant read pairs. In WES, the relative contamination levels between samples were compared by using fractions of SNVs present in non-neoplastic data with at least one mutant read. In TAS, the contamination level in terms of the percentage of neoplasm content in each non-neoplastic sample was estimated by: *(VAF_in_non-neoplastic_tissue - VAF_in_blood)/VAF_in_neoplasm* based on individual SNVs.

## Results

In the process of procuring tissue for NGS of clinical samples, we have frequently detected the presence of low level, presumptive somatic alterations in non-neoplastic tissue adjacent to the neoplasm being analyzed (Additional file 1: Figure S1). In one example, a somatic BRCA2 nonsense mutation in a breast cancer case was detected in both neoplasm and the matched adjacent non-neoplastic tissues at 23 and 2 % variant allele fractions (VAFs), respectively (Additional file 1: Figure S2). In these preliminary observations, one possible source of neoplasm contamination was the sample grossing and procurement process, during which the non-neoplastic tissue may have come into contact (directly or indirectly through operational tools) with neoplastic tissue. Although “molecular precautions” are routinely used during the histological sectioning of formalin-fixed paraffin-embedded (FFPE) samples [25], there are no corresponding standards for obtaining fresh, adjacent non-neoplastic tissue during the initial processing of tumor specimens in gross sectioning.

**Fig. 2:**
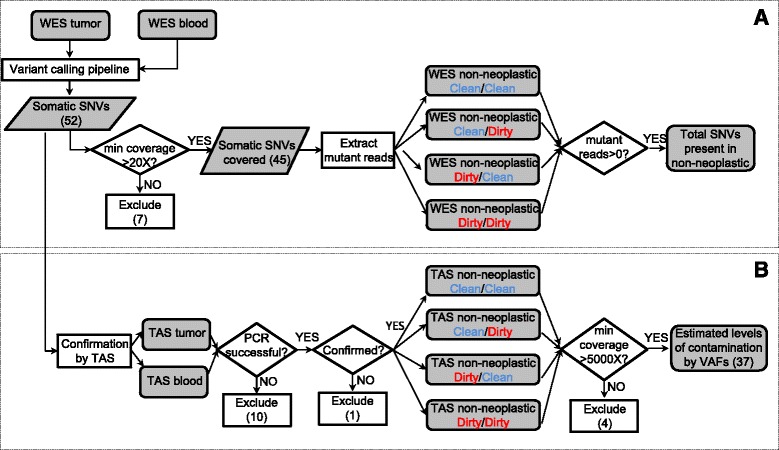
Overall flow chart of the two-stage analysis strategy. **a**, de-novo mutation calling and initial assessment of neoplasm contamination by using WES; **b**, targeted validation and ultra-deep sequencing to assess neoplasm contamination level in each adjacent non-neoplastic tissue. WES: whole exome sequencing; TAS: targeted amplicon sequencing; SNV: single nucleotide variant; VAF: variant allele fraction

Due to the high sensitivity of NGS, standard grossing procedures may lead to frequent detection of contamination in the adjacent non-neoplastic tissues, resulting in decreased somatic mutation detection sensitivity. Therefore, we evaluated two breast lumpectomies using a systematic protocol for non-neoplastic sample collection designed to determine if the level of apparent contamination can be affected by the specimen grossing and tissue procurement process (Fig. 1). The overall workflow of adjacent sample collection consists of two processes: Pathologist Assistant (PA) directed process and the Tissue Procurement (TP) process. For each of these two processes, we defined two conditions, referred to as “Clean” and “Dirty”, to denote different scenarios that presumably lead to varying levels of contamination. These collection scenarios yield four types of adjacent non-neoplastic samples (described in detail in the Methods section): “Clean/Clean”, “Clean/Dirty”, “Dirty/Clean”, and “Dirty/Dirty”. The level of contamination in adjacent non-neoplastic tissues was assessed by comparing the adjacent tissues with a blood sample from the same individual, using whole-exome sequencing (WES) followed by targeted amplicon sequencing (TAS). An analytic flow chart identifies all steps in this process (Fig. 2), which consists of three sets of sequence determination: 1) identification of *de novo* mutations in the neoplastic tissue which then served as genetic markers of neoplasm contamination; 2) confirmation of the presence of these somatic mutations in the non-neoplastic samples; 3) precise measurement of contamination levels by comparing each non-neoplastic sample to blood. The first two goals were achieved by using WES. For the third goal, we used TAS to generate the needed ultra-deep coverage.

### Identification of somatic mutations and initial assessment of tumor contamination using whole-exome sequencing

Whole exome sequencing was performed on six sample types (neoplastic, blood, and four types of non-neoplastic tissues for evaluation purpose) for each of the two patients. We generated an average of 193 million reads per sample, with mapping rate ranged from 94 to 99 %. The average coverage within the targeted region ranged from 85.6X to 279.3X in all samples, with a median of 206.3X. Most samples (10 of 12) had above 80 % of the targeted regions covered with at least 30X coverage (Additional file 3: Table S1).

Putative somatic mutations were identified by comparing neoplasm sample with the matched blood sample. In the current study, only single nucleotide variants (SNVs) were used in the estimation of neoplasm contamination. Small insertion/deletions (Indels) were excluded to avoid potential bias due to the difficulty in mapping reads harboring Indels [26]. For the current two breast carcinoma samples, we identified a total of 52 putative somatic SNVs (Fig. 2, Additional file 4: Table S2).

**Fig. 3:**
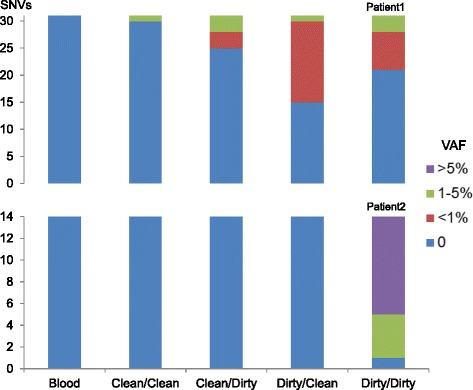
Presence of somatic SNVs in adjacent non-neoplastic tissues as the first indication of contamination. Distribution of somatic SNVs’ allele fractions (VAF) in WES data of the blood and four types of adjacent non-neoplastic tissues, classified by four categories: zero (no mutant reads detected), less than one percent, one to five percent, and greater than five percent. Y axis values represent the numbers of SNVs in each category

These SNVs were then used as markers to assess neoplasm contamination in the WES data of the four types of non-neoplastic tissue samples. A minimum 20X coverage at the mutation site in all related samples was required, retaining 45 out of the 52 SNVs, including 31 in Patient1, 14 in Patient2 (Additional file 4: Table S2). The first indication of contamination was the evidence of somatic SNVs present in non-neoplastic tissue: in Patient1, Clean/Clean: 3 % (1/31), Clean/Dirty: 19 % (6/31), Dirty/Clean: 52 % (16/31), and Dirty/Dirty: 32 % (10/31), or 33 occurrences in total, had variant allele fraction (VAF) greater than zero. Of these, eight occurrences had VAFs between 1 and 5 % and the rest were less than 1 %. Noticeably, in Patient1, one SNV was present under the condition “Clean/Clean”. Upon further manual review of the raw BAM file, this SNV in gene *CELF6* (Additional file 4: Table S2) had two mutant reads with the same read id, suggesting these two reads were from the same DNA insert (data not shown), possibly reflected artifacts during library preparation. In Patient2, none of the SNVs were present in the first three conditions (Clean/Clean, Clean/Dirty, and Dirty/Clean), but most SNVs (93 %, or 13/14, including four SNVs between 1 and 5 % and nine over 5 %) were present in the Dirty/Dirty condition (Fig. 3). No SNVs were present in the blood sample in either case. Due to the limited coverage (Additional file 3: Table S1), these results by WES should only be considered as a first indication and might not accurately reflect contamination levels.

### Accurate measurement of tumor contamination in non-neoplastic tissues using ultra-deep targeted amplicon sequencing

To obtain more precise measurements of neoplasm contamination level, ultra-deep coverage at the identified mutation sites was obtained using targeted amplicon sequencing (TAS). TAS was first performed to confirm the NGS SNVs detected in the neoplasm and matched blood samples. Ten out of 52 SNVs had PCR failure and were excluded from further analyses. For the remaining predicted SNVs, 41 of 42 were validated as somatic mutations, and one was determined to be a false positive. This false call did not affect the previous WES results (Fig. 3) because this variant was among the seven excluded variants that did not meet the minimum 20X coverage requirement as shown in the overall flow chart (Fig. 2). The 41 validated somatic SNVs were then evaluated by TAS of all four types of adjacent non-neoplastic samples. A minimum coverage of 5000X in all TAS samples was required for all SNVs; four SNVs did not meet this requirement and were excluded from further analyses. For the 37 remaining SNVs (25 in Patient1, 12 in Patient2), the median coverage of each SNV across all TAS samples ranged from 17,585X to 158,918X (Additional file 1: Figure S3).

**Fig. 4:**
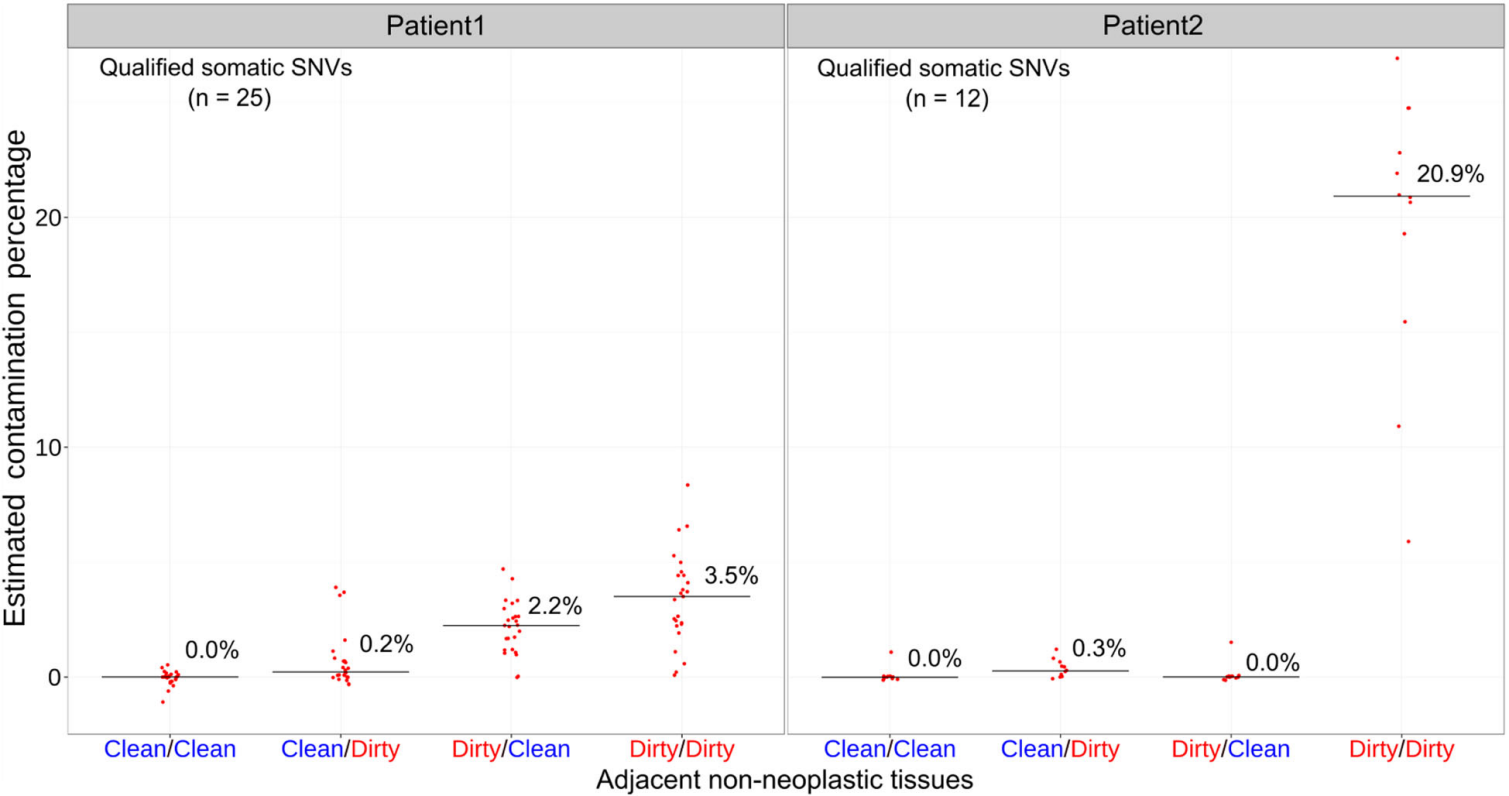
The estimated contamination levels in adjacent non-neoplastic tissues by targeted amplicon sequencing. Each *red dot* represents a previously identified somatic SNV. The contamination level was estimated by: *(VAF_in_non-neoplastic_tissue - VAF_in_blood)/VAF_in_neoplasm*. The median contamination levels for each non-neoplastic sample are plotted as a *horizontal bar* with the percentage displayed in numeric value

The allele fractions of most SNVs ranged from 10 to 30 %, and were overall consistent between the two platforms (between WES and TAS: *r* ^*2*^ = 0.8663 and 0.7443 in Patient1 and Patient2, respectively) (Additional file 1: Figure S4). The neoplasm contamination levels were estimated based on individual SNVs in all evaluated non-neoplastic samples. In Patient1, the medians of neoplasm contamination levels estimated by the 25 SNVs were 0.0, 0.2, 2.2, and 3.5 % for the four types of non-neoplastic samples (Clean/Clean, Clean/Dirty, Dirty/Clean, and Dirty/Dirty), respectively. In Patient2, based on the 12 qualified SNVs, the median contamination levels were 0.0, 0.3, 0.0, and 20.9 % (Fig. 4, Additional file 5: Table S3).

## Discussion

Tissue contamination is a well-known pitfall in pathology laboratories and tissue processing [27], and is a particular challenge for NGS-based somatic alteration analyses because of the high sensitivity of this technique. The contamination of non-neoplastic tissue with low levels of mutant nucleic acids can cause somatic mutations to be misclassified as germline events and subsequently be excluded from further analysis. Attempting to bioinformatically ‘rescue’ these missing somatic mutations is an additional challenge and may result in a high false positive rate. These problems are best solved by minimizing neoplasm contamination during sample grossing and procurement. However, until now, the relation between methods of non-neoplastic sample collection and neoplasm contamination has not been formally addressed.

In this investigation, we tried to mimic the procedures that may occur during multiple steps of non-neoplastic tissue collection. Our results provide clear evidence that neoplasm contamination in adjacent non-neoplastic samples can be reduced by optimizing the collection protocol at every step. In both tested lumpectomies, the “Clean/Clean” protocol was shown to have the lowest level of contamination, while the "Dirty/Dirty" protocol generated the highest level of contamination (Fig. 4). The lumpectomy from Patient1 had an incremental progression of increasing estimated neoplasm contamination from Clean/Clean, to Clean/Dirty, to Dirty/Clean, to Dirty/Dirty. This suggests that both “Pathologist Assistant” and “Tissue Procurement” stages could contribute to contamination. However, the specimen from Patient2 did not have such an incremental progression. Instead, there was a relatively small amount of contamination in the Clean/Dirty condition, no contamination in the Dirty/Clean condition, and very high relative contamination in the Dirty/Dirty condition. This suggests that there was no carryover of neoplasm when the specimen was initially sectioned. One possible explanation is that during the initial sectioning of the specimen, neoplastic cells were carried over mostly in the sections immediately after the neoplasm. Sampling from the less-contaminated distal portions of the specimen may explain the lack of contamination. However, since we did not keep track of the distance between the neoplasm and the tissue subsequently tested for contamination, we cannot evaluate further.

It is worth re-iterating that in the “PA dirty” condition, non-neoplastic tissue and a neoplasm section were placed in the same Petri dish to mimic the way neoplasm and non-neoplasm samples are often allowed to interact during grossing. As such, contamination that occurred during the initial sectioning of the specimen and contamination that occurred during the interaction of neoplasm and non-neoplastic tissue in the Petri dish cannot be distinguished.

Despite the fact that the neoplastic cellularity was estimated to be 50 and 70 % for Patient1 and Patient2, respectively, the VAF of the detected variants by WES and TAS tended to be significantly lower (Additional file 1: Figure S4). There are various possible explanations for this such as heterozygous status, over-representation of non-neoplastic cells, copy number changes, and heterogeneity within the neoplasm.

Another possible explanation of the presence of variants in non-neoplastic tissue is field effect. Previously, oncogenic copy number gains in common breast cancer driver genes had been found in microscopically normal breast cells outside the tumor [28]. Due to the scope of the current study, we did not assess the possibility of a field effect in a detailed fashion, which would require sampling of non-neoplastic tissues at various distance intervals from the neoplasm. Also, we did not consider the potential effect of circulating tumor cells or tumor DNA. Since the variants were not detectable under Clean/Clean condition by deep sequencing, field effect or circulating tumor cells or DNA is unlikely to be the major cause in the current study.

Most variants caused by contamination in non-neoplastic data appeared to be low frequency event (<10 %) in the current WES. Previous study on cross-patient contamination showed that as little as 2 % contaminations could cause significant increase of mutation burden due to false positives [29, 30]. For within-patient contaminations in control sample, to the best of our knowledge, there has been no systematic evaluation published on the effects on somatic mutation calling. Since algorithms have different levels of tolerance for mutations present in control, the ones with higher tolerance may lose fewer variants, but at the cost of increased false positive due to germline events. A safer approach might be excluding control samples with known contamination issues [31].

For future somatic analyses involving germline genetic material, using peripheral blood or other difficult-to-contaminate tissues as sources is a reasonable strategy. However, when grossed material is the only source of non-neoplastic sample, a specialized grossing protocol will be beneficial. First, the individual grossing the specimen should always be aware of the gross features of neoplasms. Second, clean non-neoplastic tissue should be collected from an area sectioned before the neoplasm is sectioned. Third, the non-neoplastic tissue and neoplasm/neoplasm-contaminated-tissue should be processed in separate work areas. Finally, the instruments used to cut the non-neoplastic tissue should be clean instruments that have not previously come in contact with the neoplasm.

## Conclusions

We have demonstrated that clean grossing and procurement techniques can minimize the chance of neoplasm contamination in adjacent non-neoplastic tissue, which may improve the detection of somatic mutation. Although this is a pilot study that included only two lumpectomy specimens and the analysis consisted of DNA sequencing alone, our results provide clear evidence that the methods used in sample grossing and procurement can play a non-trivial role in obtaining high quality non-neoplastic tissue samples. It is important that protocols for tissue grossing and procurement account for contamination reduction measures, thereby facilitating the acquisition of high quality, non-contaminated adjacent non-neoplastic tissues which can be used to maximize the accuracy of molecular testing.

### Availability of supporting data

The following additional data are available with the online version of this paper. “Table_S1_coverage_summary_WES.xlsx” is a table containing the mapping statistics and coverage summary of each of the 12 samples in whole-exome sequencing. “Table_S2_Somatic_SNVs_in_WES.xlsx” is a table containing all variants identified by whole-exome sequencing with coverage and VAF information; “Table_S3_VAF_by_TAS.xlsx” is a table containing the coverage, variant allele fraction and estimated contamination level for each variant by targeted amplicon sequencing; “Figures_v2.52_suppl.pptx” is a PowerPoint file contains four supplementary figures; “EVNC2.52_suppl.docx” is a word document containing the supplementary methods, description of the supplementary tables, and the legend of supplementary figures. The sequencing data from the tumors, adjacent non-neoplastic tissues and normal blood samples has been submitted to the NCBI Database of Genotypes and Phenotypes (dbGaP) under study accession [accession no. placeholder].

## Additional files

Additional file 1: Supplementary figures S1-4. **Figure S1.** An example of previous observation of neoplasm contamination in adjacent non-neoplastic tissues. **Figure S2.** An example of one somatic mutation (BRCA2S3041*) present in the matched normal data from adjacent non-neoplastic tissue. **Figure S3.** Coverage by targeted capture sequencing (TAS). **Figure S4.** Concordance of variant allele fraction (VAF) between whole-exome sequencing (WES) and targeted capture sequencing (TAS). (PPTX 925 kb)
Additional file 2: Supplementary methods, description of tables, figure legends and references. (DOCX 33 kb)
Additional file 3: Table S1. Coverage summary of whole-exome sequencing (WES). (XLSX 12 kb)
Additional file 4: Table S2. Detailed information of all somatic mutations identified by WES, including coverage, variant allele fraction (VAF) and the predicted amino acid changes. (XLSX 19 kb)
Additional file 5: Table S3. Detailed information of all somatic mutations in targeted amplicon sequencing (TAS), including coverage, VAF and estimated contamination level. (XLSX 20 kb)

## Abbreviations

Indel: Small insertion/deletion
IRB: Institutional review board
NGS: Next-generation sequencing
PA: Pathologist assistant
SNV: Single nucleotide variation
TAS: Targeted amplicon sequencing
TP: Tissue procurement
VAF: Variant allele fraction
WES: Whole-exome sequencing
