# Supplementary material for: Pitfalls of improperly procured adjacent non-neoplastic tissue for somatic mutation analysis using next-generation sequencing
Source: BMC Med Genomics. 2016 Oct 19;9:64. doi: 10.1186/s12920-016-0226-1 (PMC5070097; doi:10.1186/s12920-016-0226-1)
Supplement: Additional file 1: — Supplementary figures S1-4. Figure S1. An example of previous observation of neoplasm contamination in adjacent non-neoplastic tissues. Figure S2. An example of one somatic mutation (BRCA2S3041*) present in the matched normal data from adjacent non-neoplastic tissue. Figure S3. Coverage by targeted capture sequencing (TAS). Figure S4. Concordance of variant allele fraction (VAF) between whole-exome sequencing (WES) and targeted capture sequencing (TAS). (PPTX 925 kb) [file 12920_2016_226_MOESM1_ESM.pptx]

## Slide 1
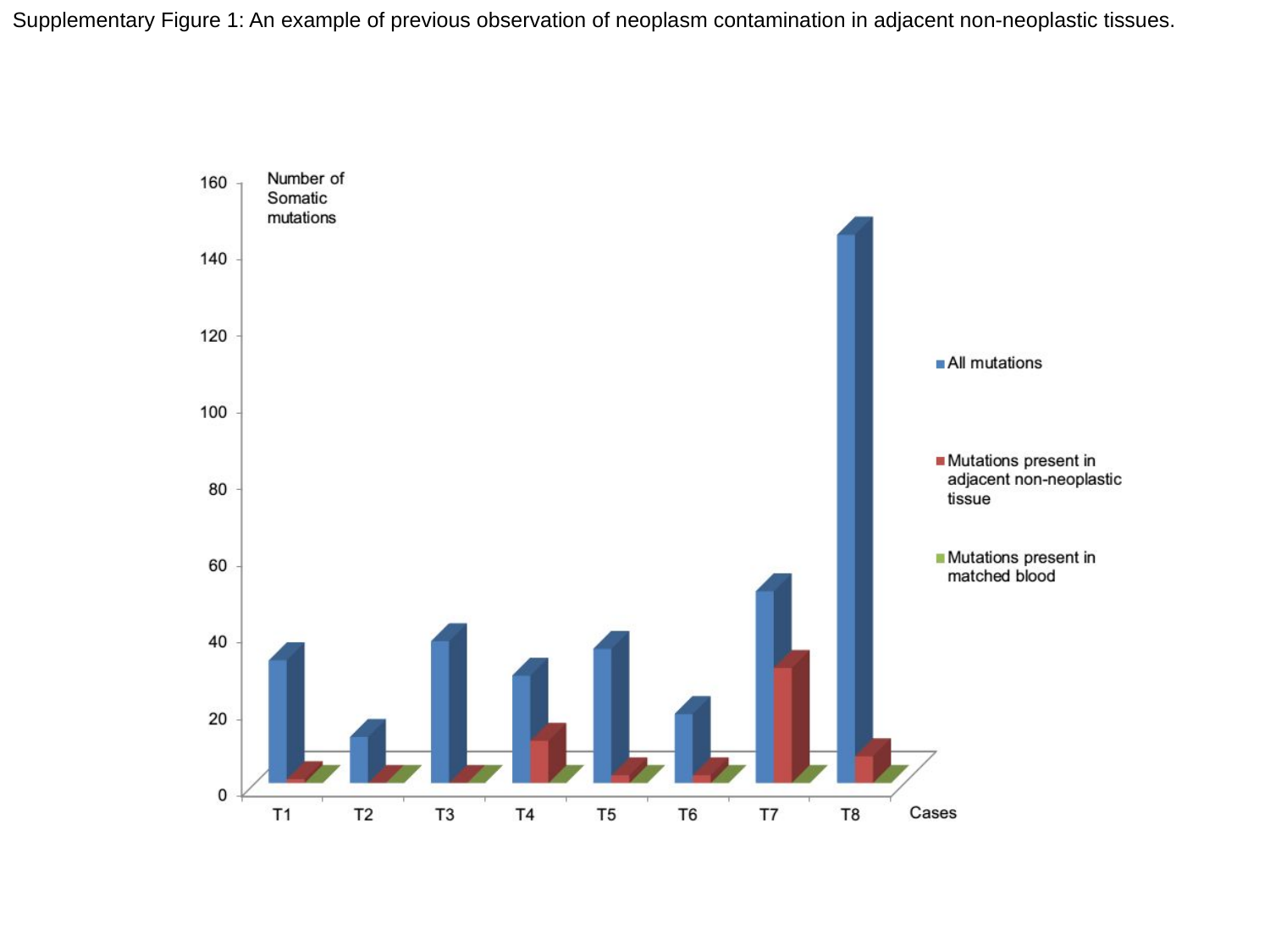

Supplementary Figure 1: An example of previous observation of neoplasm contamination in adjacent non-neoplastic tissues.

## Slide 2
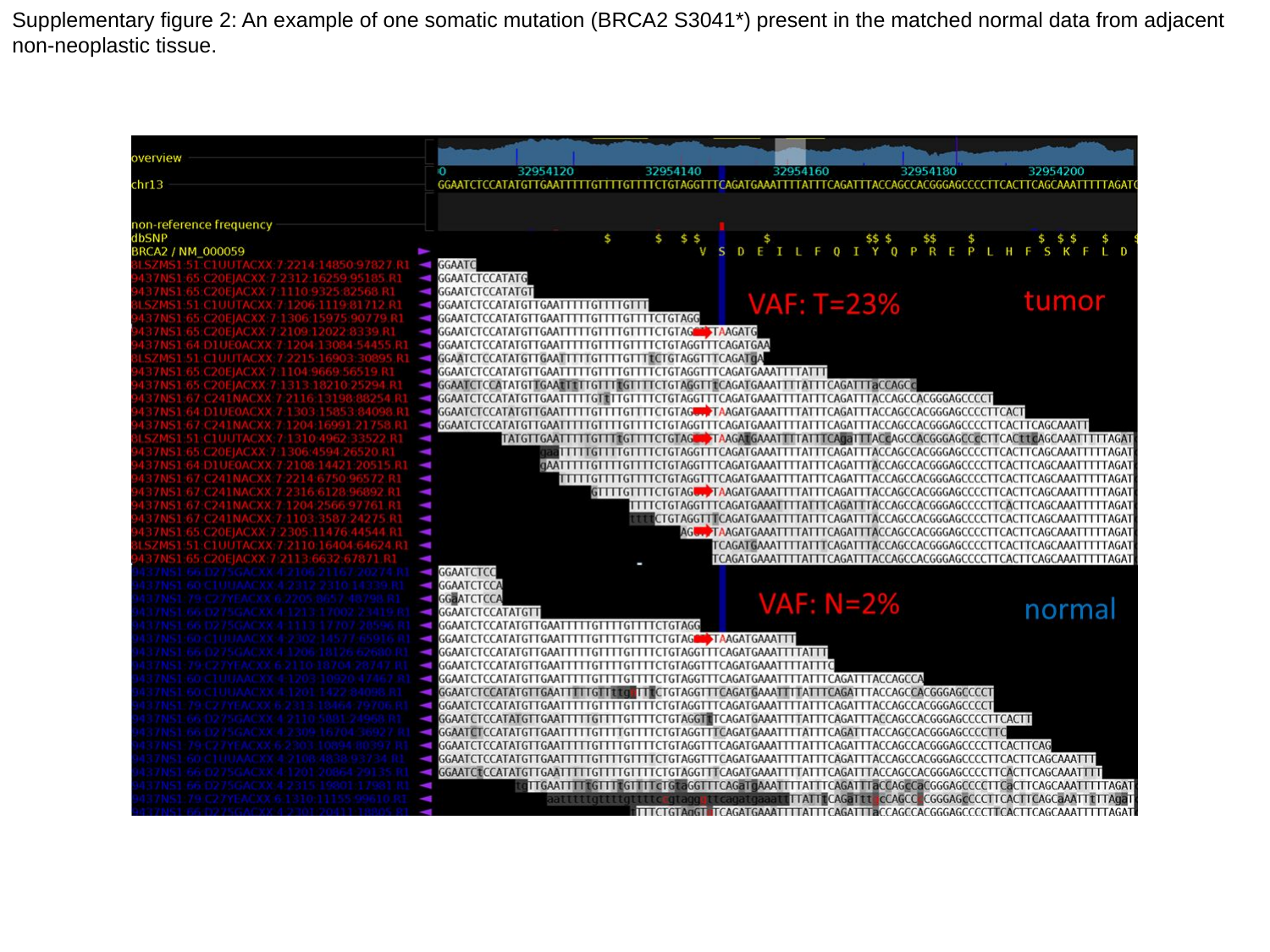

Supplementary figure 2: An example of one somatic mutation (BRCA2 S3041*) present in the matched normal data from adjacent non-neoplastic tissue.

## Slide 3
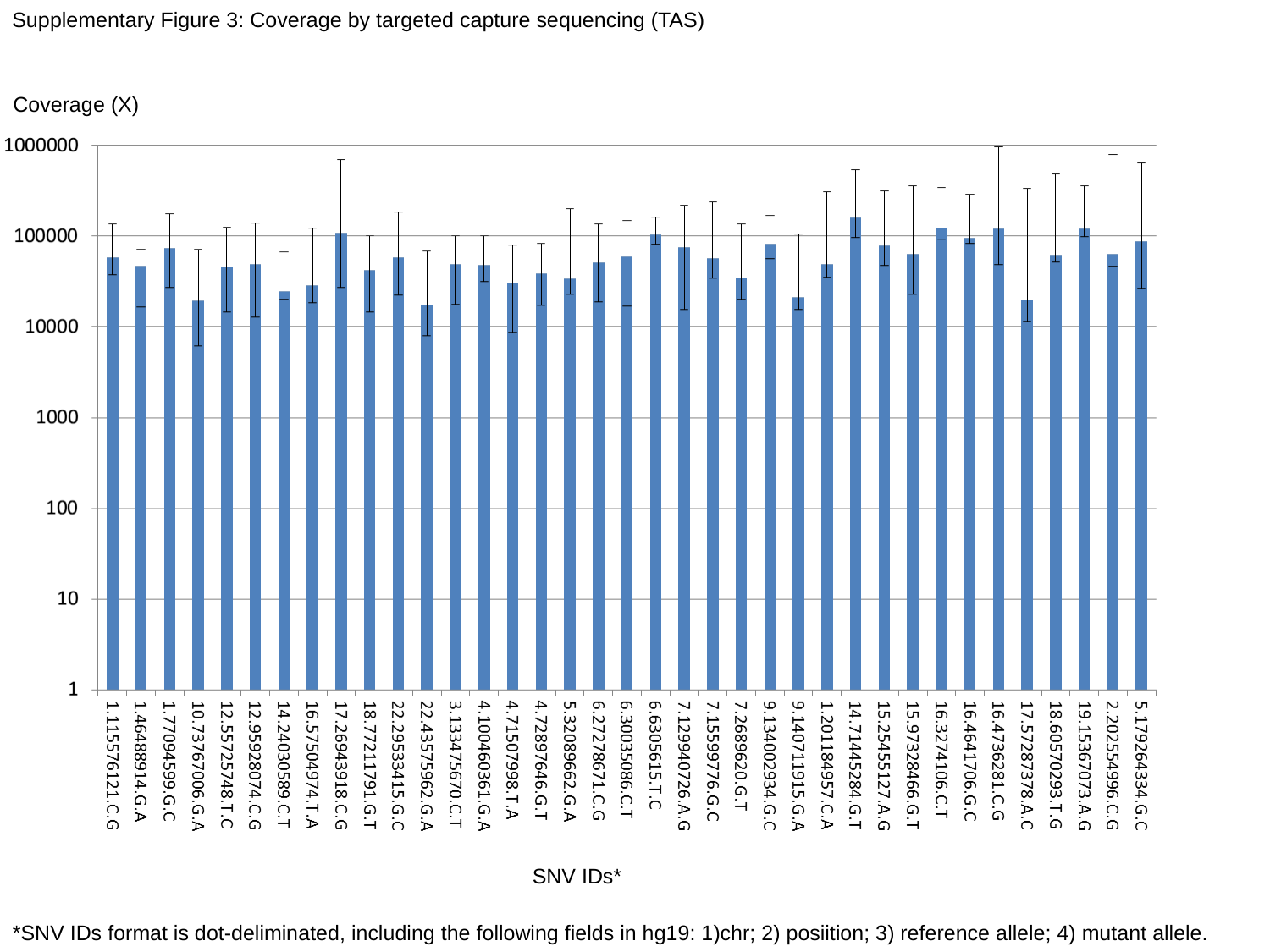

Supplementary Figure 3: Coverage by targeted capture sequencing (TAS)
Coverage (X)
SNV IDs*
*SNV IDs format is dot-deliminated, including the following fields in hg19: 1)chr; 2) posiition; 3) reference allele; 4) mutant allele.

## Slide 4
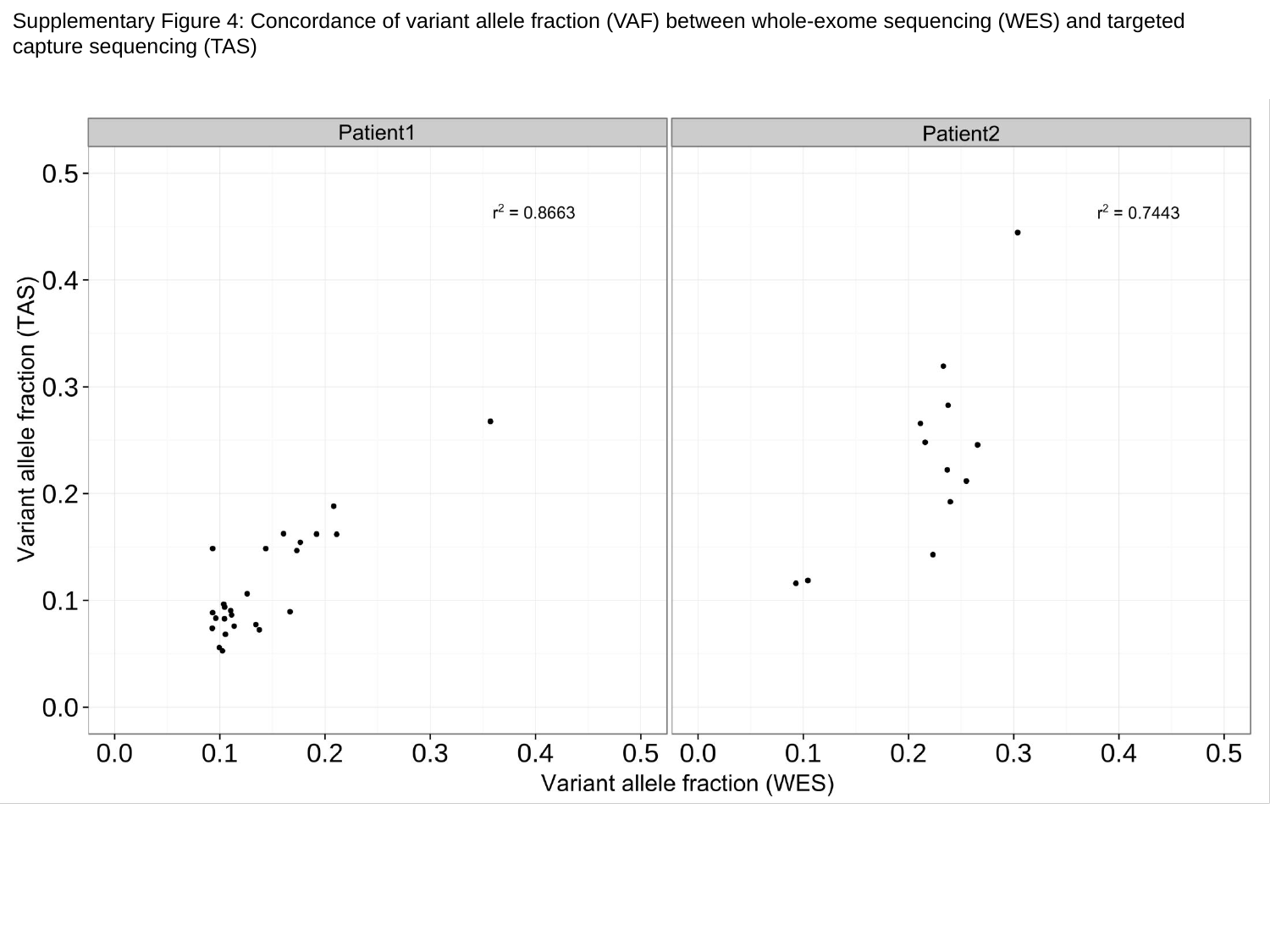

Supplementary Figure 4: Concordance of variant allele fraction (VAF) between whole-exome sequencing (WES) and targeted capture sequencing (TAS)
