# Supplementary material for: Pitfalls of improperly procured adjacent non-neoplastic tissue for somatic mutation analysis using next-generation sequencing
Source: BMC Med Genomics. 2016 Oct 19;9:64. doi: 10.1186/s12920-016-0226-1 (PMC5070097; doi:10.1186/s12920-016-0226-1)
Supplement: Additional file 2: — Supplementary methods, description of tables, figure legends and references. (DOCX 33 kb) [file 12920_2016_226_MOESM2_ESM.docx]

Supplementary information for

Pitfalls of improperly procured adjacent non-neoplastic tissue for somatic mutation analysis using next-generation sequencing

Lei Wei^1,*,#^, Antonios Papanicolau-Sengos^2,*^, Song Liu^1,*^, Jianmin Wang^1,*^, Jeffrey M. Conroy^2,*^, Sean T. Glenn^3^, Elizabeth Brese^5^, Qiang Hu^1^, Kiersten Marie Miles^2^, Blake Burgher^2^, Maochun Qin^1^, Karen Head^5^, Angela R. Omilian^5^, Wiam Bshara^5^, John Krolewski^2^, Donald L. Trump^4,6^, Candace S. Johnson^7^, Carl D. Morrison^2,#^

^1^Department of Biostatistics and Bioinformatics, ^2^Center for Personalized Medicine, ^3^Department of Cancer Genetics, ^4^Department of Medicine, ^5^Department of Pathology, ^7^Department of Pharmacology and Therapeutics, Roswell Park Cancer Institute, Buffalo, NY.

^6^Inova Dwight & Martha Schar Cancer Institute, Falls Church, VA.

^*^ L.W., A.P.S., S.L., J. W. and J.C. contributed equally to this work.

^#^ Correspondence author: [Carl.Morrison@RoswellPark.Org](mailto:Carl.Morrison@RoswellPark.Org) and [Lei.Wei@RoswellPark.Org](mailto:Lei.Wei@RoswellPark.Org)

Table of Contents

[Supplementary Methods 3](#_Toc437356463)

[Grossing and tissue procurement procedure 3](#_Toc437356464)

[DNA extraction from frozen breast tissue 4](#_Toc437356465)

[DNA extraction from blood 5](#_Toc437356466)

[Supplementary Tables 6](#_Toc437356467)

[Supplementary Table 1: Coverage summaryof whole-exome sequencing 6](#_Toc437356468)

[Supplementary Table 2: Somatic mutation de novo detection and initial contamination assessment by whole-exome sequencing 6](#_Toc437356469)

[Supplementary Table 3: Estimate neoplasm contamination level using targeted amplicon sequencing 7](#_Toc437356470)

[Supplementary Figures 8](#_Toc437356471)

[Supplementary Figure 1: An example of previous observation of neoplasm contamination in adjacent non-neoplastic tissues. 8](#_Toc437356472)

[Supplementary Figure 2: An example of one somatic mutation (BRCA2 S3041*) presents in the matched normal data from adjacent non-neoplastic tissue. 8](#_Toc437356473)

[Supplementary Figure 3: Coverage by targeted capture sequencing (TAS). 8](#_Toc437356474)

[Supplementary Figure 4: Concordance of variant allele fraction (VAF) between whole-exome sequencing (WES) and targeted capture sequencing (TAS). 8](#_Toc437356475)

[Supplementary References 9](#_Toc437356476)

# Supplementary Methods

## Grossing and tissue procurement procedure

1. Pathologist Assistant (PA) to determine if specimen is eligible for procurement based on standard procurement guidelines.
2. Eligible specimens are to be sectioned through (bread loafed) left to right using one blade for the entire specimen. PA to provide 3 tissue samples from each specimen in two separate Petri dishes:
   1. Clean non-neoplastic tissue that is sectioned through prior to slicing through the neoplasm and handled with clean blade and sterile forceps. Tissue to be placed in petri dish #1.
   2. Neoplastic tissue. Tissue to be placed in Petri dish #2.
   3. Dirty non-neoplastic tissue- tissue that is sectioned through after slicing through the neoplasm and handled with the same forceps that were used to handle the neoplastic tissue. Tissue to be placed in Petri dish #2 (along with neoplastic tissue).
3. Tissue Procurement (TP) agent to receive specimens from PA.
4. Using a clean scalpel and forceps to cut the neoplastic tissue in 300-500 mg pieces (directly in Petri dish #2 that contains one piece of non-neoplastic tissue). Snap freeze neoplastic tissue (place directly in container with LN2). Transfer tissue to cryovial once frozen. Store in -80^o^C freezer. The forceps and scalpel used to cut the neoplastic tissue will now be referred to as dirty.
5. Using a clean scalpel and forceps, cut the clean non-neoplastic tissue in half (in Petri dish #1).
   1. Take first piece of the clean non-neoplastic tissue, cut in 300-500 mg pieces using clean forceps and scalpel and place in cryovial. Place cryovial in container filled with LN2 to snap freeze. Store sample in -80^o^C freezer. This tissue is referred to as PA clean and TP clean.
   2. Take second piece of the clean non-neoplastic tissue, cut in 300-500 mg pieces using the dirty forceps and scalpel and place directly in the container with LN2 (do not place in cryovial). Transfer tissue to cryovial using the dirty forceps once frozen. Store sample in -80^o^C freezer. This tissue is referred to as PA clean and TP dirty.
6. Using a clean scalpel and forceps, cut the dirty non-neoplastic tissue in half (in Petri dish #2).
   1. Take first piece of the dirty non-neoplastic tissue, cut in 300-500 mg pieces using clean forceps and scalpel and place in cryovial. Place cryovial in container filled with LN2 to snap freeze. Label cryovial. Store sample in -80^o^C freezer. This tissue is referred to as PA dirty and TP clean.
   2. Take second piece of the dirty non-neoplastic tissue, cut in 300-500 mg pieces using the dirty forceps and scalpel and place directly in the container with LN2 (do not place in cryovial). Transfer tissue to cryovial using the dirty forceps once frozen. Label cryovial. Store sample in -80^o^C freezer. This tissue is referred to as PA dirty and TP dirty.
7. Clean cryostat by wiping the stage with 70% alcohol.
8. Obtain clean brushes that have been soaked in 100% alcohol.
9. For each patient, cut 5 QC slides from the corresponding tissues (retrieved form -80^o^C freezer).
10. QC slides to be assessed by a pathologist for presence of neoplasm (in neoplasm sections) and absence of neoplasm (in non-neoplasm sections). Samples that pass QC to be sent for DNA extractions.
11. Place samples back into -80^o^C freezer.
12. DNA is extracted following the standard DNA extraction SOP.

## DNA extraction from frozen breast tissue

Day One

1. Cut frozen tissue (up to 400 mg) with #22 scalpel and place in sterile 15 ml tube.
2. Add a total volume of 5 mL Cell Lysis Solution.
3. Aspirate multiple times with 1000 µL pipette.
4. Add 25 uL of Proteinase K to each tube.
5. Mix by inverting 25 times and incubate 55°C overnight.

Day Two

1. Add 25 uL RNase A (4 mg/mL) to the lysate.
2. Mix by inverting 25 times and incubate 15-60 minutes at 37°C.
3. Cool sample to room temperature, about 10 minutes.
4. Extract lysate with an equal volume of Phenol, Chloroform, Isoamyl alcohol (P:C:IA) (1:1).
5. Shake to mix.
6. Centrifuge at 3000 rpm for 5 minutes.
7. Using a transfer pipette, remove the aqueous layer to a clean 15 mL tube. Repeat the P:C:IA extraction. (Steps 4-7).
8. Extract the aqueous layer with an equal volume of C:IA as you did for the P:C:IA.
9. Repeat the C:IA extraction.
10. Add to the cleaned aqueous layer 0.1 volume of 2M NaOAc, pH 5.2. Note: If extracting DNA from normal breast tissue, add ~6 uL of Glycogen at this step (1 uL per 600 uL isopropanol).
11. Precipitate the DNA with 1 volume of isopropanol. The DNA will come out of solution as white strands.
12. Centrifuge at 3000 rpm for 10 minutes to pellet the DNA.
13. Remove the supernatant and wash the pellet in 70% ETOH.
14. Centrifuge at 3000 rpm for 5 minutes. Remove the supernatant and drain tubes upside down for 5-10 minutes. Upright the tubes and dry for 15-30 minutes in the BioSafety Cabinet or fume hood.
15. Add Hydration Buffer to pellets.
16. Rehydrate DNA by incubating sample for 1 hour at 65°C and overnight at room temperature.
17. Use 1.5 µL of sample on NanoDrop for concentration, 260/280 and 260/230 ratios.
18. Run 1 ug of DNA on a 1% agarose gel to confirm presence of high molecular weight DNA.

## DNA extraction from blood

DNA was extracted from blood using a Qiagen Flexigene DNA kit (Qiagen, Valencia, CA, USA) on an Autogen FlexStar extractor (Autogen Inc., Holliston, MA, USA).

# Supplementary Tables

## Supplementary Table **1:** Coverage summary of whole-exome sequencing

See Excel Table: “Table_S1_coverage_summary_WES.xlsx”

Whole exome sequencing (WES) was performed on six sample types (neoplastic, blood, and four types of non-neoplastic tissues for evaluation purpose) for each of the two patients. The mapping statistics and coverage within the targeted region were summarized for each sample.

Column definition is listed below:

A: Patient

B: Tissue type

Na1: Normal (adjacent tissue by special collection, Clean/Clean)

Na2: Normal (adjacent tissue by special collection, Clean/Dirty)

Na3: Normal (adjacent tissue by special collection, Dirty/Clean)

Na4: Normal (adjacent tissue by special collection, Dirty/Dirty)

Nb1: Normal (blood)

Td1: Tumor

C: total numbers of reads

D: percentage of reads that successfully mapped to the reference

E: percentage of reads that were marked as duplicate reads

F: average coverage within the targeted region

G to I: percentage of target regions covered by at least 10X, 20X, and 30X

## Supplementary Table **2:** Somatic mutation de novo detection and initial contamination assessment by whole-exome sequencing

See Excel Table: “Table_S2_Somatic_SNVs_in_WES.xlsx”

Somatic acquired mutations (n=52) were identified from the neoplastic tissue by comparing with the matched blood sample. Then the site of every identified mutation was checked in all four types of non-neoplastic tissues to determine the coverage and identify the presence of any mutant reads.

Column definition is listed below:

A: Patient

B: SNV: the identified SNV (hg19 chromosome, position, reference and mutant alleles)

C: gene: HUGO gene symbol

D: mRNA_acc: the mRNA accession number of the predicted transcript

E: class: the predicted type of effect of the mutation

F: aachange: the predicted amino acid change caused by the mutation

G to L: coverage in each of the 6 samples in WES

M to R: variant allele fraction (VAF) in each of the 6 samples in WES

S: whether the coverage at the mutation site is equal to or greater than 20 in all related samples

Sample naming is listed below:

Na1: Normal (adjacent tissue by special collection, Clean/Clean)

Na2: Normal (adjacent tissue by special collection, Clean/Dirty)

Na3: Normal (adjacent tissue by special collection, Dirty/Clean)

Na4: Normal (adjacent tissue by special collection, Dirty/Dirty)

Nb1: Normal (blood)

Td1: Tumor

## Supplementary Table 3: Estimate neoplasm contamination level using targeted amplicon sequencing

See Excel Table: “Table_S3_VAF_by_TAS.xlsx”

Targeted amplicon sequencing (TAS) was performed on qualified SNVs (n=37) on six sample types (neoplastic, blood and four types of non-neoplastic tissues for evaluation purpose) for each of the two patients. For each SNV, the variant allele fraction (VAF) and coverage were measured in all six sample types. The contamination level in terms of the percentage of neoplasm content in each non-neoplastic sample was estimated by: (VAF_in_non-neoplastic_tissue - VAF_in_blood) / VAF_in_neoplasm based on individual SNVs.

Column definition is listed below:

A: Patient

B: SNV: the identified SNV (hg19 chromosome, position, reference and mutant alleles)

C to H: coverage in each of the 6 samples in TAS

I to N: VAF in each of the 6 samples in TAS

O to R: contamination levels in each of the four non-neoplastic tissues estimated by (VAF_in_non-neoplastic_tissue - VAF_in_blood) / VAF_in_neoplasm

Sample naming is listed below:

Na1: Normal (adjacent tissue by special collection, Clean/Clean)

Na2: Normal (adjacent tissue by special collection, Clean/Dirty)

Na3: Normal (adjacent tissue by special collection, Dirty/Clean)

Na4: Normal (adjacent tissue by special collection, Dirty/Dirty)

Nb1: Normal (blood)

Td1: Tumor

# Supplementary Figures

## Supplementary Figure 1: An example of previous observation of neoplasm contamination in adjacent non-neoplastic tissues.

In one previous study, eight tumors with different types of non-neoplastic tissues (blood and adjacent non-neoplastic tissues) from the same patient sequenced either by whole-genome or whole-exome sequencing. The number of all identified somatic mutations, somatic mutations with mutant reads present in adjacent non-neoplastic and blood samples are indicated by blue, red and green bars. In many samples, a significant portion of all mutations were present in the “adjacent normal”, but not in the “blood normal” samples.

## Supplementary Figure 2: An example of one somatic mutation (BRCA2 S3041*) presents in the matched normal data from adjacent non-neoplastic tissue.

The raw NGS reads are displayed in Bambino viewer ([Edmonson et al. 2011](#_ENREF_1)), with mutant reads indicated by red arrows. The corresponding variant allele frequencies (VAF) were 23% and 2% in tumor and normal, respectively.

## Supplementary Figure 3: Coverage by targeted capture sequencing (TAS).

Individual SNV’s coverages across all involved samples by targeted capture sequencing were displayed using median with range across all samples in TAS.

## Supplementary Figure 4: Concordance of variant allele fraction (VAF) between whole-exome sequencing (WES) and targeted capture sequencing (TAS).

Individual SNV’s variant allele fraction (VAF) observed by WES and TAS were displayed with the Pearson’s correlation in each of the two patients.

# Supplementary References

Edmonson MN, Zhang J, Yan C, Finney RP, Meerzaman DM, Buetow KH. 2011. Bambino: a variant detector and alignment viewer for next-generation sequencing data in the SAM/BAM format. *Bioinformatics* **27**(6): 865-866.
